# Systemic identification and characterization of the conserved core NuRD complex in planarian

**DOI:** 10.3389/fragi.2025.1687668

**Published:** 2025-09-30

**Authors:** Lei Huang, Hao Wang, Shuang Wu, Jiangnan Chai, Xiaopeng Zou, Hongfei Liu, Zhengwei Guo, Yanming Wang, Yunchao Kan

**Affiliations:** ^1^ National Key Laboratory of Cotton Bio-breeding and Integrated Utilization, School of Life Sciences, Henan University, Kaifeng, Henan, China; ^2^ Department of Thoracic Surgery, Laboratory of Epigenetics and Translational Medicine, The First Affiliated Hospital of Henan University, Kaifeng, Henan, China; ^3^ School of Life Sciences, Henan Institute of Science and Technology, Xinxiang, Henan, China

**Keywords:** planarian, NuRD, histone deacetylation, differentiation, regeneration

## Abstract

The nucleosome remodeling and deacetylase (NuRD) complex, well known for its ATP-dependent chromatin remodeling and histone deacetylation activities combined in one multi-subunit complex, plays an evolutionarily conserved role in chromatin structures and gene regulation during cell growth, proliferation, and development. However, the composition and function of the NuRD complex in planarians remain incompletely unknown. Here, we identified six core components within the NuRD complex and characterized their biological roles in planarians. RNA interference (RNAi) mediated knockdown of these genes resulted in similar perturbations to both tissue homeostasis and regeneration, and the overlapping downstream genes regulated upon depletion of *MBD2/3* or *CHD4* showed similar expression alterations to that after knockdown of other NuRD complex genes, suggesting that NuRD core members may act in one complex. Additionally, the overlapping upregulated genes after depletion of NuRD complex members were expressed in neoblast and progenitor cells, among which NuRD complex core genes were enriched, suggesting transcriptional correlation between the overlapping upregulated genes and NuRD core members. Furthermore, upstream regulatory sites of the upregulated genes exhibited significant enrichment of H3K27ac, indicating the NuRD complex may deacetylate histone to modulate these genes. Notably, depletion of either *MBD2/3* or *CHD4* in planarians significantly upregulated multiple progenitor marker genes while reducing the number of somatic cells in the epidermis and intestine and downregulating multiple somatic cell marker genes, indicating that the NuRD complex may drive differentiation into somatic lineages in planarians. Collectively, our work provides a foundation to understand the essential roles of the NuRD complex in orchestrating cell differentiation, tissue homeostasis and regeneration in planarian.

## 1 Introduction

Multicellular organisms have evolved various tissue repair and regeneration strategies to cope with mechanical injuries or infections throughout their life cycles (Fu, 2021; Flora and Ezhkova, 2020; Rodrigues et al., 2019). Planarians, in particular, can regenerate entire individuals from body fragments within weeks (Morgan, 1898b; Wenemoser and Reddien, 2010; Reddien, 2018), making them an important model organism for regeneration medicine (Morgan, 1898a; Aboobaker, 2011; Dattani et al., 2019; Rossi et al., 2008; Wenemoser and Reddien, 2010; Dai et al., 2025; Pan et al., 2024). The cellular basis for planarian regeneration relies on the presence of pluripotent stem cells known as neoblasts, which are widely distributed throughout the parenchyma (Reddien, 2013; Krichinskaya and Martynova, 1975; Eisenhoffer et al., 2008). Neoblasts rapidly proliferate and migrate to the site of damage to form blastema after injury, eventually leading to the formation of complete tissues (Wenemoser and Reddien, 2010). During regeneration, epigenetic regulation is involved in stem cell proliferation, differentiation that support the restoration of missing tissue after various kinds of wounding (Hayashi et al., 2006; Wagner et al., 2011; Reddien, 2018; Rojas et al., 2024; Fraguas et al., 2021).

The nucleosome remodeling and deacetylase (NuRD) complex, a multifaceted epigenetic modulator, is integral to the developmental and physiological processes across a wide range of species, ranging from plants, nematodes and fruit flies to mammals (Hota and Bruneau, 2016; Ramírez and Hagman, 2009). This complex is characterized by its distinct biochemical properties in ATP mediated chromatin remodeling and deacetylation (Ahringer, 2000; Denslow and Wade, 2007). The NuRD complex is composed of multiple proteins, including histone deacetylases 1 and 2 (HDAC1/2), histone binding proteins 46 and 48 (RbAp46/48), chromodomain helicase DNA binding proteins 3 and 4 (CHD3/4, also referred to as Mi-2α/β), metastasis-associated proteins 1, 2, and 3 (MTA1/2/3), methyl-CpG-binding domain proteins 2 and 3 (MBD2/3), and the GATA zinc finger domain containing 2A and 2B (GATA2A/2B, also referred to as p66α/β) subunits (Allen et al., 2013; Hu and Wade, 2012; Torchy et al., 2015). In addition, it was found that lysine-specific demethylase 1 (LSD-1) also functions as a component of the NuRD complex to regulate chromatin configuration and gene expression in some contexts (Wang et al., 2009; Whyte et al., 2012; Adamo et al., 2011). The distinct histone deacetylation and chromatin remodeling activities within the complex are attributed to the unique structural and functional properties of its components (Torchy et al., 2015; Low et al., 2020; Zhang et al., 2016). The NuRD complex has been found to be implicated in vital cellular functions, such as cell signaling transduction, proliferation and differentiation, and embryonic development (Reynolds et al., 2012; Kaji et al., 2007; Ahringer, 2000; Denslow and Wade, 2007; Le Guezennec et al., 2006; Kaji et al., 2006; Yoshida et al., 2008; McDonel et al., 2009). However, the composition and functional significance of the NuRD complex in planarian biology remains further study.

Here, we identified and characterized the complement of core genes of NuRD complex in planarian: First, after silencing individual NuRD complex genes by means of RNAi, we observed very similar phenotypes of regeneration abnormality, tissue homeostasis disruption and ultimate animal lysis. Second, RNA-seq data analyses reveal a shared set of downstream genes regulated upon depletion of *MBD2/3* or *CHD4*, with similar changes observed after knockdown of other NuRD complex genes. Third, using published single-cell RNA-seq (scRNA-seq) data, we found that the downstream upregulated overlapping genes were enriched in neoblasts and progenitor cells where the NuRD complex genes were also highly expressed. Fourth, genome-wide chip-seq showed that the upregulated overlapping genes have a significant enrichment of H3K27ac modification within their promoter regions, indicating that the NuRD complex may derepress them through histone deacetylation. Collectively, the above results indicate that the NuRD complex may affect both homeostasis and regeneration through regulating these shared genes in planarian. Additionally, after depletion of *MBD2/3* or *CHD4*, marker genes of neoblast remain relatively stable expression, whereas the expression of marker genes of multiple progenitor cells significantly increased, and marker genes of intestine and epidermal somatic cells decreased, suggesting that the NuRD complex may drive the differentiation from progenitor cells to somatic cells during homeostasis in planarian. Taken together, our work provides a foundation for further insights into epigenetic regulation through NuRD complex during tissue homeostasis and regeneration in planarian and other animals with regenerative capacity.

## 2 Materials and methods

### 2.1 Phylogenetic analysis of the NuRD complex

The NuRD complex protein sequences of 15 species were downloaded from the NCBI database, including *Homo sapiens*, *Mus musculus*, *Drosophila melanogaster* and *Caenorhabditis elegans*. The protein sequences were subjected to multiple sequence alignment using the muscle software with default parameters (Edgar, 2021). The phylogenetic tree was constructed using the RAxML software (Stamatakis, 2014). Additionally, the tree was drawn using the ggtree function in R.

### 2.2 Experimental animal preparation

Animals used in all experiments were asexual strain of the planarian *Schmidtea mediterranea* and were fed in formulated seawater (1.6 mM NaCl, 1.0 mM CaCl_2_, 1.0 mM MgSO_4_, 0.1 mM MgCl_2_, 0.1 mM KCl, and 1.2 mM NaHCO_3_ in Milli-Q water) at 20 °C. Animals were fed weekly with beef liver. Before all the experiments, animals ∼5 mm in length were starved for 1 week.

### 2.3 Gene cloning

The NuRD complex genes were identified from the dd_smed_g4 genome version (Supplementary Figure S1) (Grohme et al., 2018). Target genes were PCR amplified from a cDNA library. PCR products were used as templates for RNA probe synthesis and dsRNA production (see primer sequences in Supplementary Table S5).

### 2.4 RNA interference

All the dsRNAs were synthesized using an *in vitro* transcription reaction kit (Promega, P1700) from PCR-generated DNA templates with flanking T7 promoters, ethanol precipitated, and annealed after resuspension in RNase free ddH_2_O. The concentrations of dsRNA were measured using NanoDrop (Thermo Fisher). Then 4 μg dsRNA was mixed with 20 μL planarian food (beef liver) to feed 20 animals in a 6 well dish. RNAi treated animals were fed a total of 4 times, with each feeding spaced 2 days apart. At 9 days post RNA interference (dpi), quantitative reverse-transcription PCR (qRT-PCR) analyses of gene expression levels, whole-mount *in situ* hybridization, immunofluorescence and TUNEL staining were performed. For regeneration experiments, animals were amputated into head, trunk, and tail fragments at 8 dpi. For all RNAi feeding experiments, GFP dsRNA was used as a control.

### 2.5 Quantitative reverse-transcription PCR

To evaluate the knockdown efficiency and assess the relative RNA expression level, we conducted qRT-PCR assays. All RNA samples were digested with TURBO™ DNase. cDNA synthesis was performed using the RevertAid™ First Strand cDNA Synthesis Kit (Invitrogen, 18064014). TB Green^®^ *Premix Ex Taq*™ II (Takara, RR820A) was used according to the manufacturer’s instructions to perform qRT-PCR using a CFX96 Real-Time PCR System (Bio-Rad). GADPH was used as an internal control. p values were calculated using the two-tailed unpaired Student’s t-test. The primers used for qRT-PCR were listed in Supplementary Table S5.

### 2.6 Bulk RNA sequencing and analyses

*MBD2/3* RNAi treated animals were placed in Trizol (Life Technologies, 15596018), and then frozen at −80 °C. After thawing, animals were resuspended in Trizol by pipetting until dissolved and total RNA was isolated using the Trizol standard isolation protocol from Life Technologies. mRNA is enriched using the mRNA Capture Beads. After purification with beads, the mRNA is fragmented using high temperatures then is used as a template to synthesize the first strand cDNA in a reverse transcription enzyme mixture system. After synthesizing the second strand cDNA, end repair and A-tailing were completed, adapters were ligated and Hieff NGS^®^ DNA Selection Beads were used for purification to select target fragments. Then PCR library amplification was performed and sequencing was carried out using the Illumina Novaseq X Plus.

Fastp (version 0.23.4) was used to clean low quality reads and Bowtie2 (version 2.2.8) was used to remove ribosome RNA reads (Langmead and Salzberg, 2012). Paired-end clean reads were mapped to the dd_smed_g4 genome using HISAT (version 2 2.1.0) and other parameters were set as the default (Kim et al., 2015). For each transcribed region, a FPKM (fragment per kilobase of transcript per million mapped reads) value was calculated to quantify its expression abundance and variations using RSEM (Li and Dewey, 2011). Then gene counts were used as input to identify differentially expressed genes in R package DESeq2 with cutoff values of read counts >10, FDR <0.1, and |log_2_ Fold Change| > 0.8. For generating the heatmap plot, FPKM or RPKM values were used as input.

*CHD4* RNAi sequencing raw data were obtained from the GEO database (GSE72389) (Tu et al., 2015). Methods to analyze the sequencing data were the same as analyzing the *MBD2/3* RNAi data.

### 2.7 GO, KEGG and GSEA enrichment analyses

The GO term and the KEGG annotation of each gene were obtained using the eggnog-mapper website (http://eggnog-mapper.embl.de/) by protein blast. Then constructed an OrgDb R package using R script. GO term and KEGG enrichment analyses were performed by the enrichGO or enricher function of the clusterProfiler package, and the top categories were displayed using the lollipop plot. GSEA was performed by the GSEA function of the clusterProfiler package (Yu et al., 2012).

### 2.8 Histology and TUNEL staining

For TUNEL staining, animals were subsequently fixed in 4% paraformaldehyde overnight at 4 °C, dehydrated in graded sucrose solution (10%/20%/30%). Fixed specimens were embedded in paraffin and sectioned at 8 μm thickness. TUNEL staining of tissue sections was performed using the One Step TUNEL Apoptosis Assay Kit (Beyotime, C1090).

### 2.9 Whole-mount *in situ* hybridizations

Whole-mount fluorescent *in situ* hybridizations were performed using a protocol similarly as previously described (King and Newmark, 2013; Forsthoefel et al., 2014). RNA probes were synthesized using the DIG RNA labeling kit (Roche, 11175025910). Planarians were treated with 5% N-acetylcysteine in PBS for 10 min, followed by fixation in 4% formaldehyde in PBSTx for 20 min. The animals were sequentially washed twice in PBSTx and 50% methanol for 5 min, and once with 100% methanol for 10 min before storage at −20 °C. Then animals were bleached for 3 h in a bleaching solution containing 5% formamide, 0.5×SSC, and 1.2% hydrogen peroxide on a light source. After washing once with 1xSSC and twice with PBSTx for 5 min each, animals were treated with 10 μg/mL proteinase K in PBSTx for 10 min. After re-fixation in 4% formaldehyde for 20 min and washing twice with PBSTx and once with prehybridization buffer for 10 min each, animals were hybridized with DIG labeled probes at 56 °C for 18 h. Following hybridization, animals were washed twice in wash-hybridization buffer, thrice in 2 × SSC, four times in 0.2 × SSC, twice in TNTx. Subsequently, animals were blocked with 5% horse serum in TNTx for 2 h. The animals were incubated with the DIG antibody conjugated with Alkaline Phosphatase 1:1,000 diluted in blocking solution overnight at 4 °C. Then animals were washed 6 times with TNTx for 20 min each. The color was subsequently developed using NBT and BCIP (1:100) in development solution for 30 min to 3 h. Subsequently, animals were washed twice with PBSTx for 5 min each, once in 100% ethanol for 20 min and resuspended in PBSTx. Brightfield images were taken with a Nikon Stereo Microscope and processed with the NIS element software and the Adobe Photoshop software.

### 2.10 Single-cell RNA-seq data analyses

The scRNA transcriptome data with the accession number CRA007941 of eight regeneration time points in planarian *Schmidtea mediterrane*a were downloaded from https://ngdc.cncb.ac.cn/gsa/browse/CRA007941. Sequence reads were aligned to the dd_smed_g4 genome using CellRanger to generate gene expression matrices that were further analyzed using Seurat. The merge function was used to merge sample matrices. Low-quality cell data from the raw data with nFeature_RNA lower than 500 or higher than 6,000 were filtered, then data with nCount_RNA lower than 2,000 or higher than 50,000 were further filtered. Remaining high-quality data were further processed using NormalizeData, FindVariableFeatures and ScaleData to generate normalized expression values. The top 2,000 highly variable genes were used for the RunPCA analyses. The first 30 PCs were used to construct a shared nearest neighbor graph and further generate the two-dimensional UMAP embeddings. The resolution was set as 1 in the FindClusters function to generate clusters. After removing double cells using DoubletFinder, the FindAllMarkers function was used to calculate the differential gene expression of clusters and samples. Finally, we used marker genes in Supplementary Table S3 to annotate different cell types. For the genes that need to be displayed, we used the AddModuleScore and Featureplot function to calculate gene expression and generate plots. For the GSEA analyses of different cell stages, we used the FindAllMarkers function to calculate the differential gene expression.

### 2.11 ChIP-seq data analyses

The ChIP-seq with the accession numbers PRJNA832235 (H3K27ac) and PRJNA338116 (H3K27me3, H3K4me1 and H3K4me3) in planarian *S. mediterranea* were downloaded from the NCBI databases. The reads were quality-checked with the fastp software. The reads were aligned to the dd_smed_g4 genome with Bowtie2 and duplicated reads were removed with Samtools. Peaks were called with MACS2 with default parameters, and annotated with ChIPseeker package and plotted with Gviz package in R.

### 2.12 Statistical analyses

Data are expressed as the mean ± the standard deviation of three independent experiments. *p*-values less than 0.05 were considered statistically significant. All statistical analyses were performed in the GraphPad Prism 9 software.

## 3 Results

### 3.1 NuRD complex genes are dynamically and coordinately expressed during development and regeneration in planarian

**FIGURE 1:**
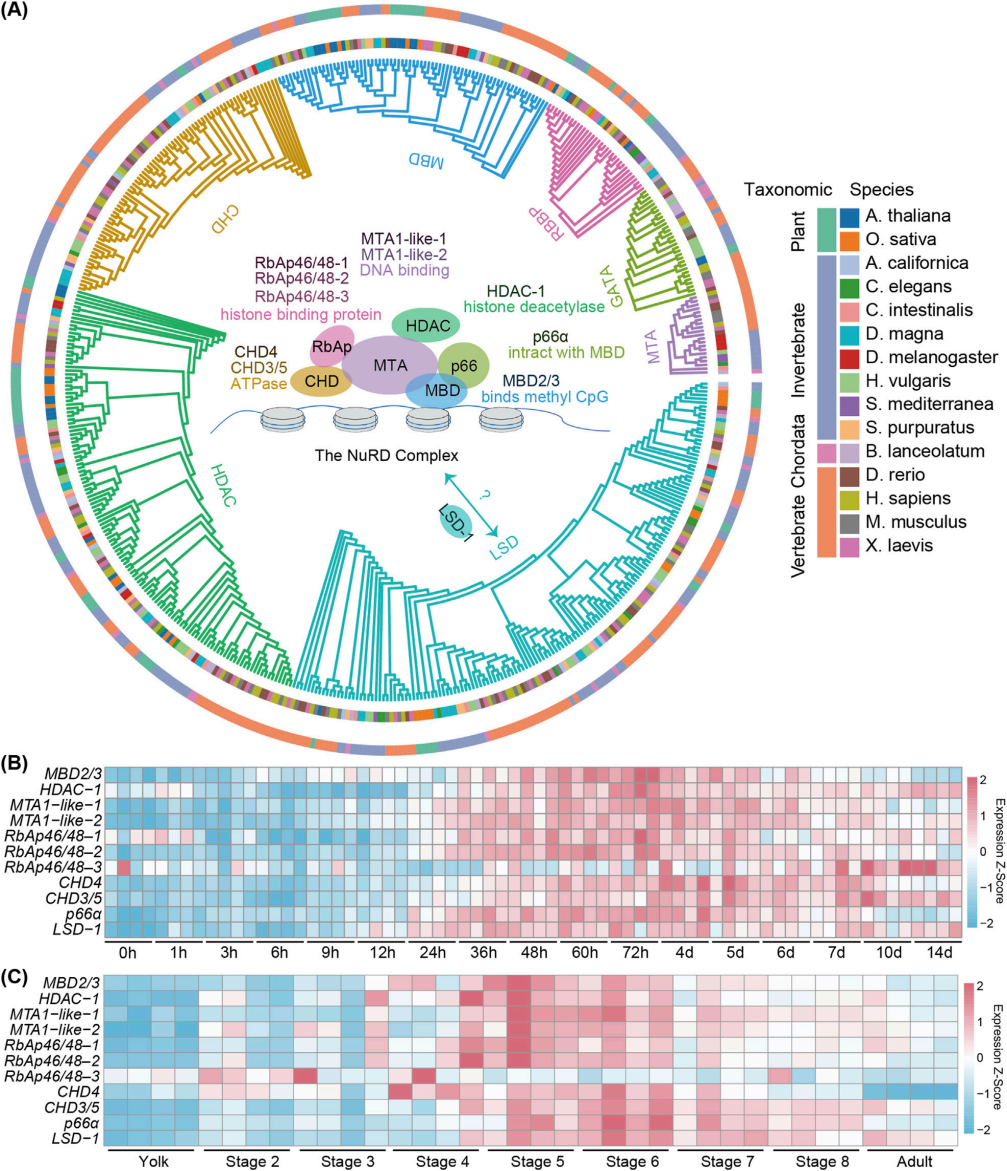
The evolutionary conservation and the spatiotemporal expression pattern of planarian NuRD complex genes. **(A)** Maximum likelihood (ML) phylogenetic tree depicting the evolutionary relationships of 11 genes forming the NURD complex across 15 species. Orthologous genes, species names and their taxonomic genera are denoted by colors. **(B)** Heatmap showing the expression profiles of NuRD complex genes at different time points during planarian regeneration. Color code denotes the expression values (z-score). **(C)** Heatmap showing the expression profiles of NuRD complex genes at different stages of development. Color code denotes the expression values (z-score).

To investigate the NuRD complex function in planarians, we searched the planarian genome and identified 10 transcripts encoding its putative six core components, along with an ortholog of the *LSD-1* gene (Figure 1A; Supplementary Figure S1). Comprehensive phylogenetic analyses across 15 species, including plants, invertebrates and vertebrates, revealed evolutionary conservation of these NuRD complex genes (Figure 1A; Supplementary Figure S1A). To determine the temporal expression pattern of these genes in planarians, we analyzed the expression of the NuRD genes using the published RNA-seq datasets covering planarian regeneration and development (Davies et al., 2017; Zeng et al., 2018; Grohme et al., 2018). In regenerating lateral tissue fragments excised from planarian body walls, we observed a coordinated upregulation of NuRD complex genes from 24 h to 6 days post-amputation (dpa) (Figure 1B). As tissue remodeling approached completion from 7 dpa and later on, except that the expression of *HDAC-1*, *CHD3/5*, and *LSD-1* remained stable relative to earlier time points and *RbAp46/48–3* was significantly upregulated (Figure 1B), the expression of the remaining NuRD components progressively declined (Figure 1B).

From RNA-seq results at different stages of development (Davies et al., 2017), we found that the expression of the NuRD complex genes was coordinately increased from stages 4–6 during which the formation of major organs occurs, while decreased from stage 7 to adults when organ formation completes (Figure 1C), suggesting the NuRD complex likely plays a role in tissue formation and organ patterning. Interestingly, *RbAp46/48–3* exhibited a divergent expression pattern distinct from other NuRD complex genes during both development and regeneration (Figures 1B,C), suggesting that *RbAp46/48-3* may not act as a core structural component of the NuRD complex.

### 3.2 The NuRD complex genes are essential for tissue homeostasis and regeneration in planarian

**FIGURE 2:**
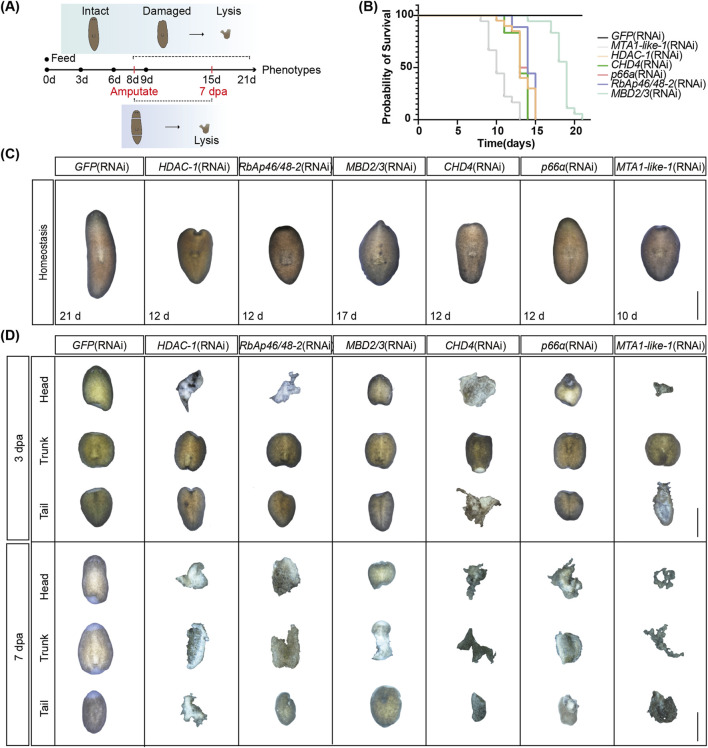
NuRD complex core genes regulate tissue homeostasis and regeneration. **(A)** Working schemes of the RNAi experiment in planarians. **(B)** Survival curves of planarians after RNAi treatment of the six core NuRD complex genes (*n* = 18). **(C)** Images of tissue homeostasis after RNAi treatment of the six core NuRD complex genes (*n* = 18). **(D)** Images of regenerating planarians after RNAi treatment of the six core NuRD complex genes (*n* = 18). Scale bars: 1,000 µm.

To examine the function of the NuRD complex in planarians, we used RNAi to knock down the 11 NuRD complex genes (Figure 2A). Except for *CHD3/5*, 10 out of the 11 NuRD complex genes were depleted ranging from 50% to 90% (Supplementary Figure S2A). Depletion of eight genes in adults (e.g., *HDAC-1* and *MBD2/3*) led to animal lysis and decreased the viability of planarians (Figures 2B,C and Supplementary Figures S2B,C), indicating that these genes are essential for adult tissue homeostasis. The lysis started at the head and neck regions of the animals and subsequently progressed to the tail region before complete lysis (Figure 2C; Supplementary Figure S2C). Notably, animals lysed significantly earlier after *MTA1-like-1* RNAi treatment than any other gene depletion. This might be due to multiple roles of *MTA1-like-1* in NuRD complex formation (Millard et al., 2014; Alqarni et al., 2014) and in RNA binding and mitotic progression (Li et al., 2023; Liu et al., 2020). In contrast, depletion of *LSD-1* and *RbAp46/48–3* bore a minimal impact on the normal living and survival of planarians, implying that these factors are nonessential for the integrity and function of the core NuRD complex to sustain homeostasis (Supplementary Figures S2B,C).

To determine how NuRD affects regeneration, we amputated the head and tail of planarians at 9 days after RNAi treatment (Figure 2A). Depletion of core NuRD components—*HDAC-1*, *RbAp46/48–1/2*, *MBD2/3*, *CHD4*, *p66α*, and *MTA1-like-1/2*—which are essential for adult tissue homeostasis, led to a complete failure in blastema formation and anterior/posterior regeneration by 3 dpa, progressing to widespread tissue lysis between 3-7 dpa (Figure 2D; Supplementary Figure S2D). This functional congruence indicates that depletion of core components in the NuRD complex disrupts the integrity and activity of the complex and ultimately abolishes the regenerative capacity of planarians. For *LSD-1* and *RbAp46/48–3*, nonessential for adult tissue homeostasis (Supplementary Figure S2C), the blastema was smaller and the eye formation was delayed at 7 dpa in animals with *LSD-1* RNAi, whereas *RbAp46/48–3* depletion did not induce obvious regeneration defects (Supplementary Figure S2D), suggesting these two genes may not be essential in the NuRD complex both in regeneration and homeostasis.

Based on the expression profiles and phenotypes after RNAi, we selected six genes, including *MBD2/3*, *CHD4*, *RbAp46/48–2*, *HDAC-1*, *MTA1-like-1*, and *p66α*, representing each of the essential core components of the NuRD complex for further functional studies.

### 3.3 Common NuRD complex downstream target genes identified by transcriptomic analyses

**FIGURE 3:**
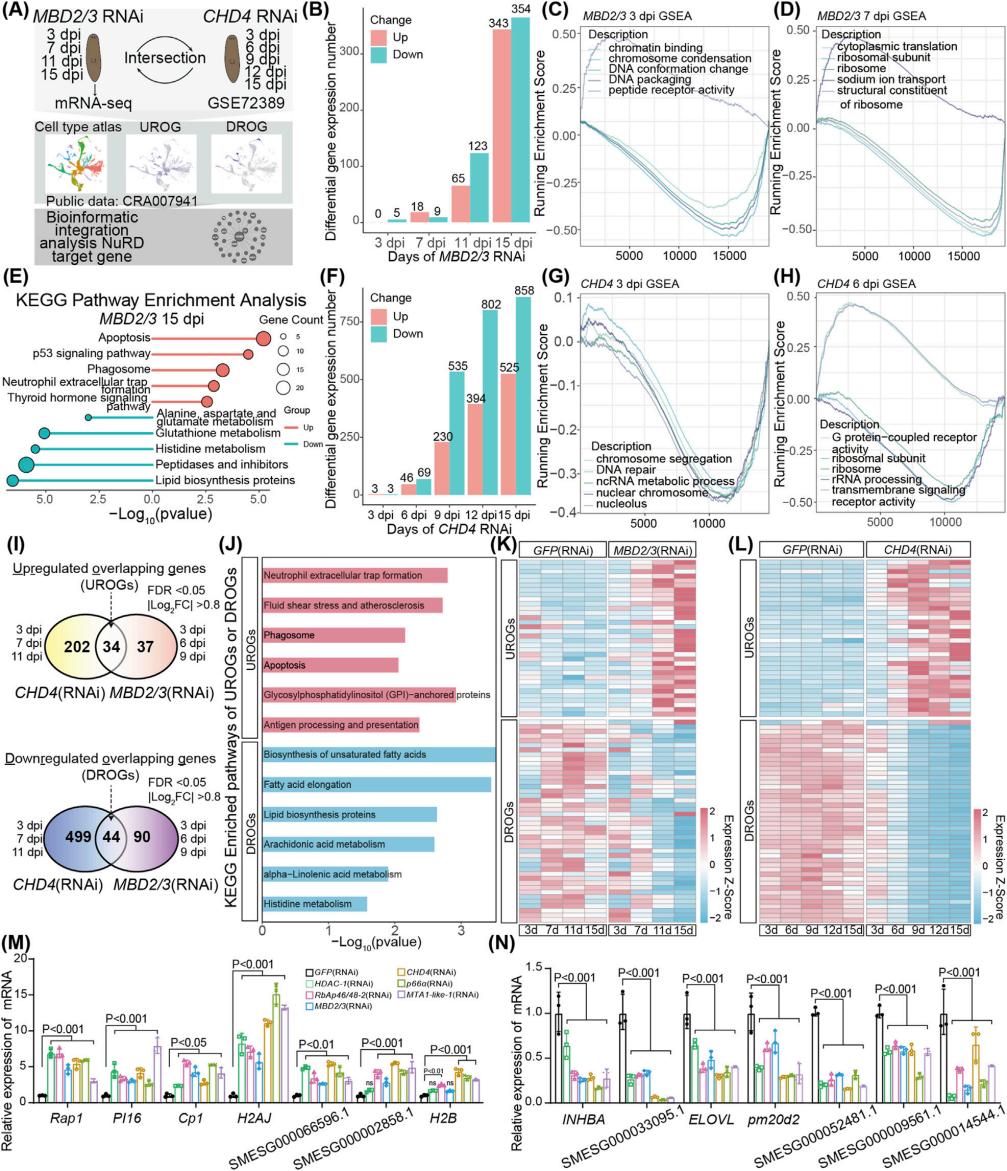
Transcriptomic analyses identified common NuRD target genes. **(A)** Schematic of RNA-seq experiment after *MBD2/3* RNAi treatment and integrated transcriptome data analyses after *MBD2/3* and *CHD4* depletion. **(B)** Bar plot showing the number of differential expression genes after *MBD2/3* RNAi treatment. **(C,D)** Gene set enrichment analyses of gene expression changes at 3 and 7 dpi after *MBD2/3* depletion. **(E)** KEGG pathway enrichment analyses of significantly differentially expressed genes at 16 dpi after *MBD2/3* treatment. **(F)** Bar plot showing the number of differential expression genes after *CHD4* depletion. **(G,H)** Gene set enrichment analyses of gene expression changes after *CHD4* depletion at 3, 6 and 9 dpi. **(I)** Venn diagram showing the UROGs and DROGs across the first three time points after *MBD2/3* and *CHD4* depletion. **(J)** KEGG pathway enrichment analyses of 34 UROGs and 44 DROGs. **(K,L)** Heatmaps showing the expression profiles of 34 UROGs and 44 DROGs. Color code denotes the expression values (z-score). **(M,N)** qRT-PCR analyses of 7 UROGs **(M)** and 7 DROGs **(N)** after NuRD complex gene depletion. Error bars represent mean values ±SD (*n* = 3). A two-way ANOVA was used for statistical comparisons.

To analyze the effect of the NuRD complex on transcription in planarians, we first chose *MBD2/3* for further studies. We fed adult animals with *MBD2/3* RNAi and performed RNA-seq at 3, 7, 11, and 15 dpi treatment (Figure 3A). The depletion of *MBD2/3* led to altered expression of 5 and 27 genes at 3 and 7 dpi, respectively (Figure 3B; Supplementary Table S1). The number of genes with altered expression increased to 188 and 697 at 11 and 15 dpi, respectively (Figure 3B; Supplementary Table S1). In total, there were 388 upregulated and 470 downregulated genes identified from the four treatment time points (Supplementary Figures S3A,B). Gene Set Enrichment Analyses (GSEA) showed that the expression of the chromatin condensation and DNA packaging gene sets decreased at 3 dpi (Figure 3C), cytoplasmic translation and ribosomal subunits gene set expression was significantly declined at 7 dpi (Figure 3D), while DNA packaging and ribosome gene sets showed decreased expression at 11 dpi (Supplementary Figures S3C–E). Furthermore, some cell type specific marker genes, such as the intestinal marker gene gst-3 showed significant decrease at 15 dpi, while the neoblast marker gene *H2AJ* and the epidermal progenitor marker gene *EGR4* were upregulated (Supplementary Figure S3F). Kyoto Encyclopedia of Genes and Genomes (KEGG) analyses showed that the 343 upregulated genes at 15 dpi were enriched in the apoptosis, phagosome, and the p53 signaling pathway (Figure 3E, upper). The 354 downregulated genes at 15 dpi are involved in the peptidases and inhibitors, lipid biosynthesis proteins, and amino acid metabolic pathways (Figure 3E, lower). Consistent with the increase in the apoptotic genes, TUNEL staining of tissue sections showed the number of TUNEL positive cells was significantly increased near the epidermal area in 15 dpi animals (Supplementary Figure S3G).

To identify the common set of genes regulated by the NuRD complex genes in planarians, we performed bioinformatic analyses of the published *CHD4* RNAi transcriptome data (Tu et al., 2015), using the similar criteria as the *MBD2/3* analyses for better comparison. Similar to the *MBD2/3* RNAi treatment, the number of genes affected by *CHD4* depletion gradually increased from 3, 6, 9, 12 to 15 dpi (Figures 3F,H,I). GSEA analyses showed the nuclear chromosome and the chromosome segregation pathway gene expression was decreased at 3 dpi (Figure 3G), the ribosome subunits and rRNA processing pathway genes were decreased at 6 dpi (Figure 3H), the fatty acid metabolic process and the cellular amino acid metabolic process pathway gene sets showed decreased expression at 9 dpi (Supplementary Figure S3J), and the DNA-binding transcription activator activity and RNA polymerase II transcription regulatory region sequence-specific DNA binding gene sets were reduced at 12 dpi (Supplementary Figure S3K). KEGG enrichment analyses showed the 525 upregulated genes at 15 dpi were enriched in the neuroactive ligand−receptor interaction, adherens junction, and apoptosis pathways, while the 858 downregulated genes were enriched in the transporters, lysosome, and amino acid metabolic pathways (Supplementary Figures S3L–M). Taken together, the above results demonstrated a high similarity in downstream pathway changes after *MBD2/3* RNAi and *CHD4* RNAi treatment, suggesting that the NuRD complex genes regulate similar downstream gene pathways to impact on planarian biology.

To identify genes co-regulated by the NuRD complex genes, we first conducted an intersection analysis of pooled differentially expressed genes affected by both *CHD4* or *MBD2/3* at their respective first three time points after RNAi treatment. We identified 34 upregulated overlapping genes (UROGs), and 44 downregulated overlapping genes (DROGs) (Figure 3I; Supplementary Table S2). KEGG pathway enrichment analyses revealed that the UROGs were enriched in neutrophil extracellular trap formation (mainly chromatin component genes such as *H2AJ* and *H2B*), fluid shear stress and atherosclerosis, phagosome, and apoptosis pathways (Figure 3J), while the DROGs were enriched in metabolic pathways including lipid biosynthesis (e.g., biosynthesis of unsaturated fatty acids and fatty acid elongation) and histidine metabolism (Figure 3J). Moreover, 103 UROGs and 162 DROGs were identified from the 15 dpi *MBD2/3* and the 12 and 15 dpi *CHD4* treatment groups (Supplementary Figures S4A,B). Gene expression heatmap showed the 34 UROGs and 44 DROGs identified from the three early time points kept their expression change trend at later time points after RNAi treatment (Figures 3K,L), revealing that these are core genes downstream of *CHD4* or *MBD2/3*. To analyze whether the NuRD complex member genes regulate the expression of the same set of genes, we depleted *MBD2/3*, *CHD4*, *RbAp46/48–2*, *HDAC-1*, *MTA1-like-1*, and *p66α* by RNAi, respectively. qRT-PCR analyses showed that these genes exhibited similar effects on UROGs (e.g., *PI16*, *Cp1* and *H2AJ*) and DROGs (e.g., *INHBA*, *ELOVL* and *pm20d2*) (Figures 3M,N). These results support our notion that a common set of genes are coordinately regulated by core NuRD components in planarian.

### 3.4 Depletion of NuRD complex disrupts progenitor cell differentiation

**FIGURE 4:**
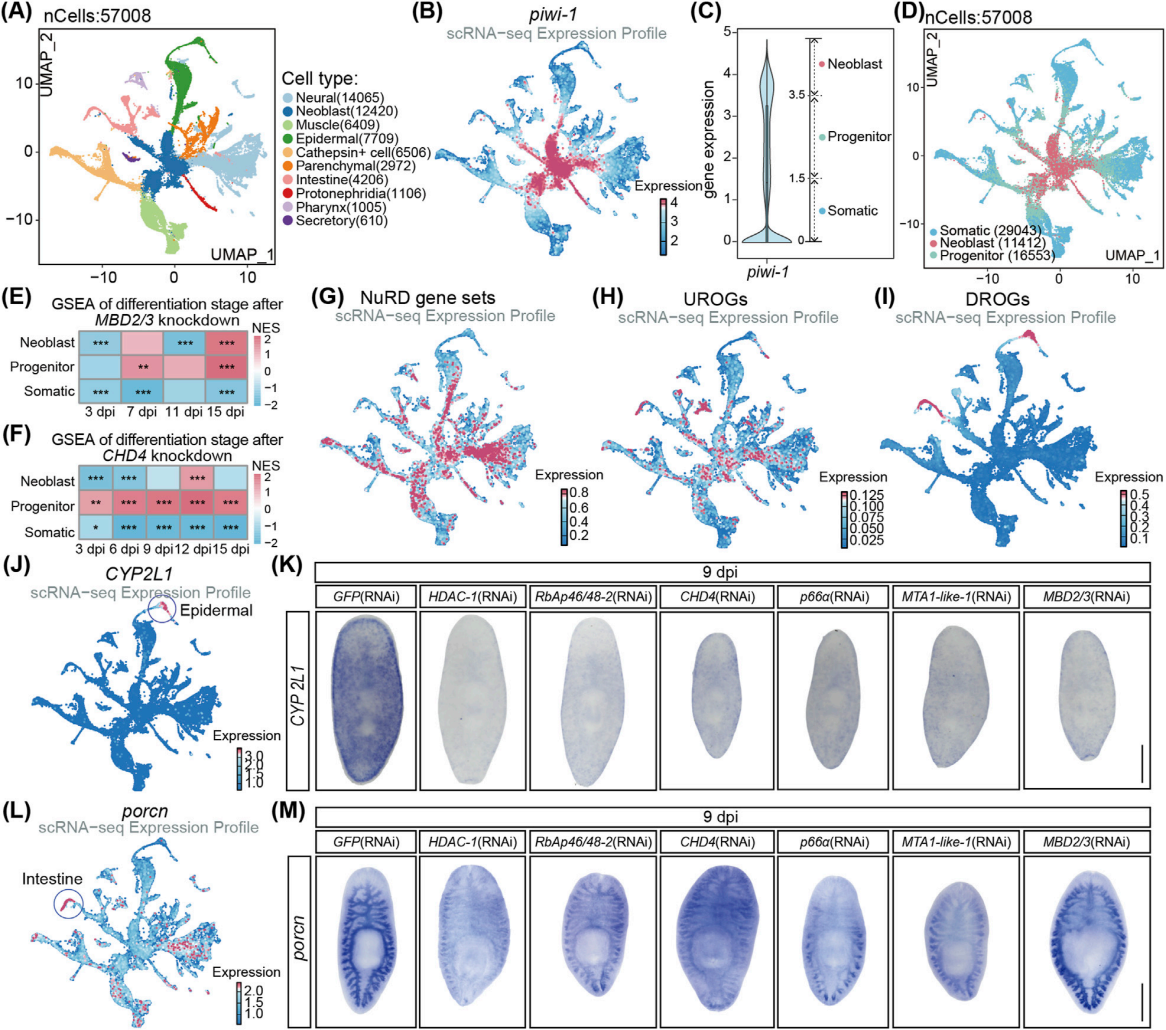
The NuRD complex regulates cell differentiation in planarians. **(A)** UMAP visualization of published scRNA-seq data, color coded by cell types. **(B)** Feature plot of *piwi-1* expression. **(C)** Violin plot showed *piwi-1* expression in whole planarian cells and classified as 3 cell types. **(D)** UMAP visualization of the classified cell types. **(E,F)** Differentiation stage signature scoring analyses following *MBD2/3* knockdown **(E)** and *CHD4* knockdown **(F)**. The permutation test was used for statistical comparisons, ^*^ *p* < 0.05, ^**^ *p* < 0.01, ^***^ *p* < 0.001. **(G)** Feature plot of NuRD complex gene set expression pattern. **(H,I)** Feature plot of the 34 UROGs **(H)** and the 44 DROGs **(I)** expression pattern. **(J)** Feature plot of *CYP2L1* expression. **(K)** WISH images of *CYP2L1* expression after the depletion of NuRD complex core genes. **(L)** Feature plot of porcn expression. **(M)** WISH images of porcn expression after the depletion of NuRD complex core genes. Scale bar: 500 µm.

ScRNA-seq analysis is a powerful tool to denote the 10 major cell types based on the expression of the commonly accepted cell type marker genes in planarians (e.g., epidermal and intestine) (Figure 4A; Supplementary Table S3) (Cui et al., 2023). For example, *piwi-1* ^*+*^ cells denote the neoblasts in the UMAP diagram of cell types (Figure 4B). Moreover, based on the expression levels of *piwi-1*, cells were classified into three differentiation stages including: neoblasts, progenitors and somatic cells (Figures 4C,D). To investigate the role of NuRD in cell differentiation, we performed GSEA analyses for the transcriptome after *MBD2/3* and *CHD4* RNAi treatment by using the respective marker genes of the three differentiation stages. The progenitor cell stage had the highest normalized enrichment score (NES) and some progenitor marker genes were significantly upregulated after *MBD2/3* or *CHD4* RNAi treatment (Figures 4E,F; Supplementary Figure S5). By contrast, the somatic cell stage had the lowest NES and some somatic marker genes were significantly downregulated in both transcriptomes (Figures 4E,F; Supplementary Figure S5). Neoblasts cell stage genes were the most downregulated at early RNAi treatment time points, while slightly recovered at later time points (Figures 4E,F; Supplementary Figure S5). Moreover, the progenitor cell maker genes were much upregulated while the somatic cell marker genes were decreased after *MBD2/3* and *CHD4* depletion, indicating the NuRD complex likely regulates the progenitor to somatic cell differentiation process.

ScRNA-seq analyses found the six essential core NuRD complex genes were expressed mainly in neoblasts and progenitor cells (Figure 4G; Supplementary Figures S4C–H), where the 34 UROGs were predominantly expressed as well (Figure 4H; Supplementary Figures S4I–K), suggesting that NuRD complex likely directly represses the expression of these genes during differentiation. In contrast, DROGs were mainly enriched in epidermal and intestine somatic cells lacking the expression of NuRD complex (Figure 4I; Supplementary Figures S4L–N), suggesting that the decrease in DROGs likely resulted from the disruption of differentiation and formation of epidermal and intestine cells after the NuRD genes depletion.

To further analyze the effect of the NuRD complex depletion on cell differentiation, we performed whole mount *in situ* hybridization (WISH) on the cell type marker genes, including *piwi-1* for NB cells, *prog-1* for early epidermal progenitor cells, *agat-1* for late epidermal progenitor cells, *CYP2L1* for epidermal somatic cells and *porcn* for intestine somatic cells (Figures 4B,J,L; Supplementary Figures S6A,B) (Tu et al., 2015; Scimone et al., 2010; Fincher et al., 2018). At 9 dpi, *piwi-1* ^*+*^ cells showed no significant changes (Supplementary Figure S6C), and qRT-PCR detected no alterations in *piwi-1* expression after the NuRD gene depletion (Supplementary Figure S6D), suggesting that NuRD complex is not essential for the maintenance of neoblasts. For the epidermal lineage genes, the *prog-1* expression was increased at the pre-pharyngeal head area in the WISH assay and in qRT-PCR analyses (Supplementary Figures S6E,F), *agat-1* expression was decreased at the head area in the WISH assay and in qRT-PCR analyses (Supplementary Figures S6G–H), while somatic epidermal marker gene *CYP2L1* expression was drastically decreased in the whole animals (Figure 4K). Moreover, intestine somatic cell marker gene *porcn* was significantly decreased in WISH analyses (Figure 4M). Taken together, these results demonstrate that the NuRD complex is dispensable for neoblast self-renewal but essential for differentiation into intestinal and epidermal somatic cells during homeostasis.

### 3.5 The NuRD complex may maintain homeostasis through histone deacetylation in planarian

**FIGURE 5:**
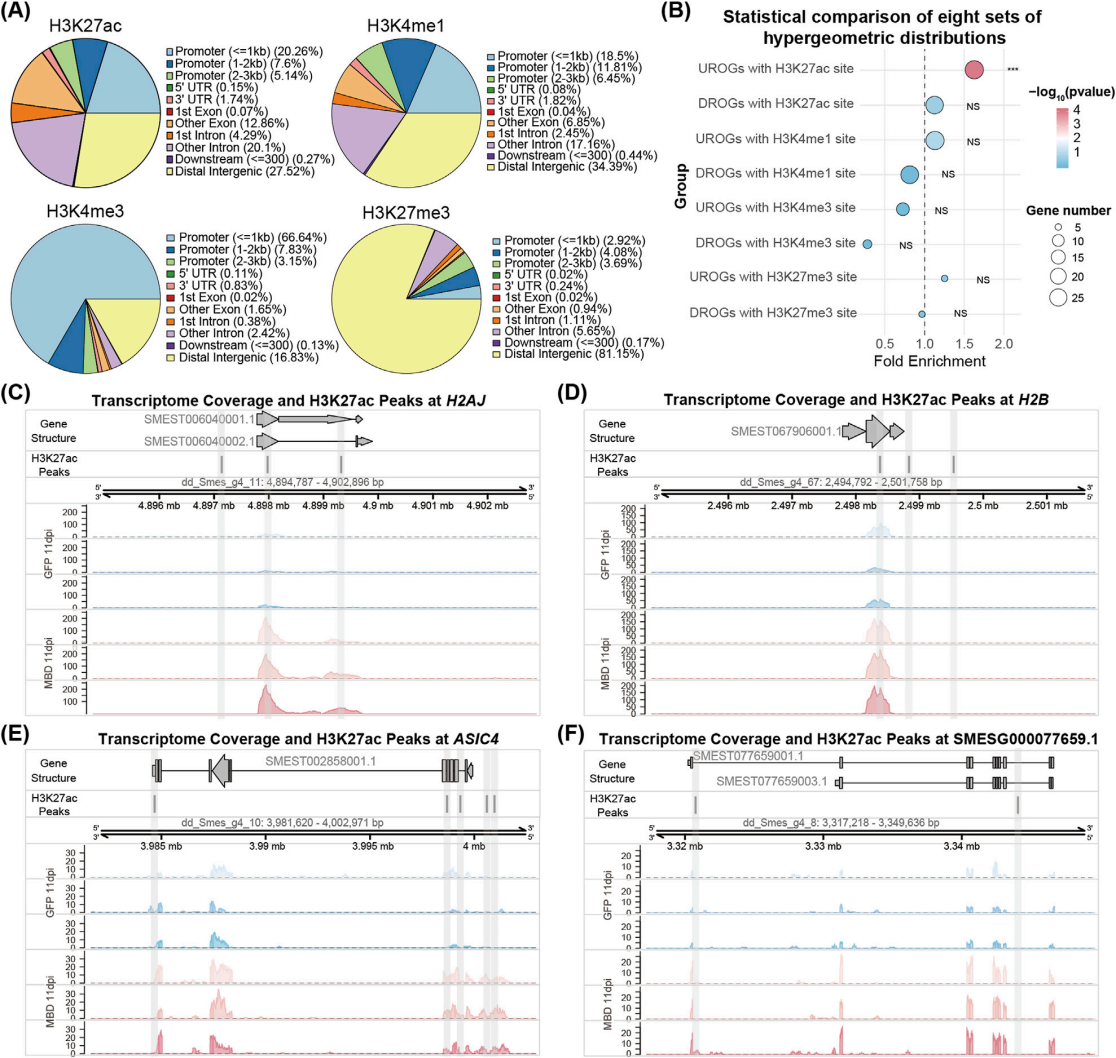
Genomic profiling of histone acetylation and methylation mediated regulation. **(A)** Pie chart of peak distribution in different genomic regions of four histone modifications. **(B)** Dot plot showing hypergeometric distribution analyses of 34 UROGs and 44 DROGs with H3K27ac, H3K4me1, H3K4me3, and H3K27me3 modification sites. **(C–F)** The genomic tracks showing the distribution of RNA-seq reads at 11 dpi MBD2/3 treatment and H3K27ac peaks across four UROGs (*H2AJ*, *H2B*, *ASIC4* and SMESG000077659.1).

To analyze the effects of the NuRD complex on histone modifications, we analyzed previously generated ChIP-seq data using the H3K27ac, H3K27me3, H3K4me1 and H3K4me3 antibodies (Neiro et al., 2022; Mihaylova et al., 2018). H3K4me1 was enriched at promoter and distal intergenic regulatory regions, H3K4me3 was enriched at promoter regions, and H3K27me3 was enriched at distal intergenic regulatory regions in planarians (Figure 5A). Approximately 33% of the H3K27ac sites were enriched at promoter regions in planarians (Figure 5A). Differential genomic region enrichment of above modifications supports their specialized roles in epigenetic regulation of the genome. Hypergeometric analyses of histone modifications revealed H3K27ac peaks in the vicinity of 29/34 UROGs, with 28 genes with peaks specifically occupying promoter regions (Figure 5B; Supplementary Figure S7A; Supplementary Table S4), representing a statistically significant enrichment of H3K27ac at UROGs regulatory regions (*p* = 5.94 × 10^−5^), such as *H2AJ*, *H2B*, *ASIC4* and SMESG000077659.1 (Figures 5C–F). In contrast, 26/44 of DROGs contain H3K27ac peaks, with no significant promoter enrichment of H3K27ac in DROGs (*p* = 0.23) (Figure 5B; Supplementary Figure S7A; Supplementary Table S4). Notably, there are no significant enrichment of three histone methylation modifications (H3K27me3, H3K4me1 and H3K4me3) in the upstream regions of UROGs or DROGs (Figure 5B; Supplementary Figure S7A). Taken together, the strong promoter association of H3K27ac suggests that UROGs might be derepressed through histone deacetylation after the depletion of NuRD complex genes.

## 4 Discussion

In this study, we identified and characterized the NuRD complex in planarians, revealing its coordinated dynamic expression during development and regeneration. RNAi screening identified 8 components (e.g., *HDAC-1*, *CHD4*, and *MBD2/3*) essential for tissue homeostasis and regeneration, and 3 non-essential components. We selected *MBD2/3* knockdown for mechanistic studies due to its higher survival rate *versus* other essential subunits, enabling extended observation. To study the NuRD complex’s direct target network, we focused on early stages after *CHD4* and *MBD2/3* depletion, and the transcriptome data from three early time points revealed 78 shared genes regulated by *CHD4* and *MBD2/3* (Figure 3I). Additionally, qRT-PCR analyses showed consistent changes across all six NuRD complex genes depletions, indicating these genes could be a core set of downstream targets that mediate the function of NuRD complex in planarians. Among the differentially expressed genes, 34 UROGs with upstream regulatory sites exhibiting higher levels of H3K27 acetylation are enriched in the same cell population as the NuRD complex genes, suggesting that NuRD complex may regulate these genes through histones deacetylation. For the 44 DROGs enriched in the somatic cells, their decreased expression likely reflects that these somatic cells cannot form properly after the NuRD complex disruption. Moreover, after *MBD2/3* or *CHD4* depletion, multiple progenitor cell marker genes were significantly upregulated, while somatic cell marker genes were repressed. These results indicate that proper NuRD-mediated deacetylation is required to drive stem cell differentiation during tissue homeostasis in planarians. Such epigenetic regulation advances our understanding of how dynamic chromatin states orchestrate stem and somatic cell fate decisions during normal life cycles and regeneration.

Tissue lysis was a fierce phenotype that occurred in the depletion of multiple house-keeping genes, such as *bruli1*, *vasa1*, and *hsp90* (Wagner et al., 2012; Reddien et al., 2005; Dong et al., 2018). We found that knocking down core components of NuRD complex led to a striking tissue lysis and hence reduced animal survival and regeneration abnormality after amputation in planarians, suggesting the essential role of NuRD complex in tissue homeostasis and regeneration. Using identical RNAi methods, we successfully depleted 10 out of the 11 NuRD complex genes, but not *CHD3/5*. *CHD3/5* is expressed at low levels from transcriptome data (Zeng et al., 2018), which likely contributed to its lack of depletion with RNAi. The role of epigenetic regulatory genes associated with the NuRD complex in planarians was reported in several prior studies (Scimone et al., 2010; Jaber-Hijazi et al., 2013; Robb and Sánchez Alvarado, 2014; Hubert et al., 2015; Vásquez-Doorman and Petersen, 2016; Dattani et al., 2019). It was found that *CHD4* gene is mainly expressed in neural cells and is required for stem cell differentiation in planarian (Scimone et al., 2010), and *MBD2/3* is necessary for pluripotent stem cell differentiation independently of DNA methylation (Jaber-Hijazi et al., 2013). Moreover, *p66α* is needed for eye and epidermal differentiation (Vásquez-Doorman and Petersen, 2016), whereas MTA homologous genes are essential for the uniform distribution of adult pluripotent stem cells in *Dugesia japonicais* (Sato et al., 2022). However, previous studies mainly focused on the investigation of individual genes, rather than the coordinated regulatory mechanisms as a whole complex (Vásquez-Doorman and Petersen, 2016; Scimone et al., 2010; Jaber-Hijazi et al., 2013; Sato et al., 2022). Here, we demonstrated the cellular localization, molecular regulation, and primary functions of the NuRD complex. Our study established that the NuRD members function as an integrated complex to regulate the downstream genes through histone deacetylation and as a critical “gatekeeper” that licenses progenitor differentiation, thereby directing tissue homeostasis and regeneration.

In mammals, the NuRD complex suppress gene transcription by altering chromatin structure through synergistic actions of histone deacetylase subunits HDAC1/HDAC2 and the ATP-dependent nucleosome remodeler subunit Mi2 (Zhang et al., 1999; Saito and Ishikawa, 2002; Milstone et al., 2020). In addition, the NuRD complex exerts its function through associating with the methylated DNA via the MBD3 and MBD2 subunits. MBD2 directly binds to methylated CpG sites and recruits the NuRD complex to methylated regions to inhibit transcription through histone deacetylation and nucleosome remodeling (Baubec et al., 2013; Le Guezennec et al., 2006). Previous studies found that there was extremely low DNA methylation in the planarian genome and MBD2/3 were unable to function through binding to the methylated DNA (Jaber-Hijazi et al., 2013). Consistently, there is no significant enrichment of three histone methylation modifications (H3K27me3, H3K4me1 and H3K4me3) in the upstream regions of UROGs or DROGs in our study, suggesting that the NuRD complex may regulate gene expression by histone deacetylation in planarians.

The failure to establish the *in vitro* cell culture system in the planarian limits cellular-level studies of physical interactions between the NuRD complex components. Moreover, there are no corresponding antibodies to the NuRD complex components available, we could not perform biochemical validation of direct interactions between distinct NuRD complex subunits. Further study is needed to biochemically characterize the NuRD components involved in homeostasis and regeneration in planarian. Additionally, it is worth investigating whether the NuRD complex also modulates regenerative processes in other animals with high regenerative capacity, such as *salamanders*. If validated, a key mechanistic question is whether species-specific adaptations in the NuRD complex—including distinct structural configurations, specialized subunit isoforms, or regulatory histone post-translational modification sites—support the maintenance of expansive stem cell pools with potent self-renewal and differentiation capacities in highly regenerative species. Furthermore, it is also interesting to determine whether these species-specific NuRD features could be used to enhance or reprogram stemness in mammalian cells in future studies.

The NuRD complex is well established as a key regulator of cellular aging, with its functional loss leading to blocked differentiation, proteostatic stress, and apoptosis—processes integral to aging across multiple systems (Horikawa, 2020; Kruta et al., 2021; Panier et al., 2024; Rando et al., 2025; Ulfig and Jakob, 2024). Planarians have recently emerged as a compelling model for aging research, exhibiting conserved hallmarks such as telomere shortening, decreased fertility, motility reduction, tissue architecture alterations, and elevated oxidative stress (Tan et al., 2012; Dai et al., 2025). Notably, our findings demonstrate that knockdown of NuRD in planarians recapitulates aging-like phenotypes, supporting a conserved role for NuRD dysfunction in promoting aging processes. Furthermore, evidence that regeneration and neoblast activation can reverse aging features (Dai et al., 2025) underscores the importance of epigenetic regulation, a process through which the NuRD complex likely modulates aging in planarians.

Taken together, our study provides foundational evidence for the evolutionarily conserved function of NuRD in mitigating aging through epigenetic control of tissue renewal. By leveraging the planarian, we not only solidify its position as a powerful model for aging research but also open new avenues for exploring conserved epigenetic pathways that govern stem cell biology, regeneration, and aging across diverse species.

## Data Availability

The datasets presented in this study can be found in online repositories. The names of the repository/repositories and accession number(s) can be found in the article/Supplementary Material.
