# Supplementary material for: Systemic identification and characterization of the conserved core NuRD complex in planarian
Source: Front Aging. 2025 Sep 30;6:1687668. doi: 10.3389/fragi.2025.1687668 (PMC12518275; doi:10.3389/fragi.2025.1687668)
Supplement: Supplementary file 3 [file Supplementaryfile1.docx]

Supplementary Figures for “Systemic Identification and Characterization of the Conserved Core NuRD Complex in Planarian”

Lei Huang ^1†^, Hao Wang ^1†^, Shuang Wu ^1^, Jiangnan Chai ^1^, Xiaopeng Zou ^1^, Hongfei Liu ^1^, Zhengwei Guo ^2^, Yanming Wang ^2*^, and Yunchao Kan ^1,3*^

^1^National Key Laboratory of Cotton Bio-breeding and Integrated Utilization, School of Life Sciences, Henan University, Kaifeng 475004, Henan, China

^2^Department of Thoracic Surgery, Laboratory of Epigenetics and Translational Medicine, The First Affiliated Hospital of Henan University, Kaifeng, Henan, 475000, China

^3^School of Life Sciences, Henan Institute of Science and Technology, Xinxiang 453003, Henan, China

^†^These authors contributed equally to this work and share first authorship

*** Correspondence:**Yanming Wang
yanmingwang@henu.edu.cn；

Yunchao Kan

yckan@henu.edu.cn


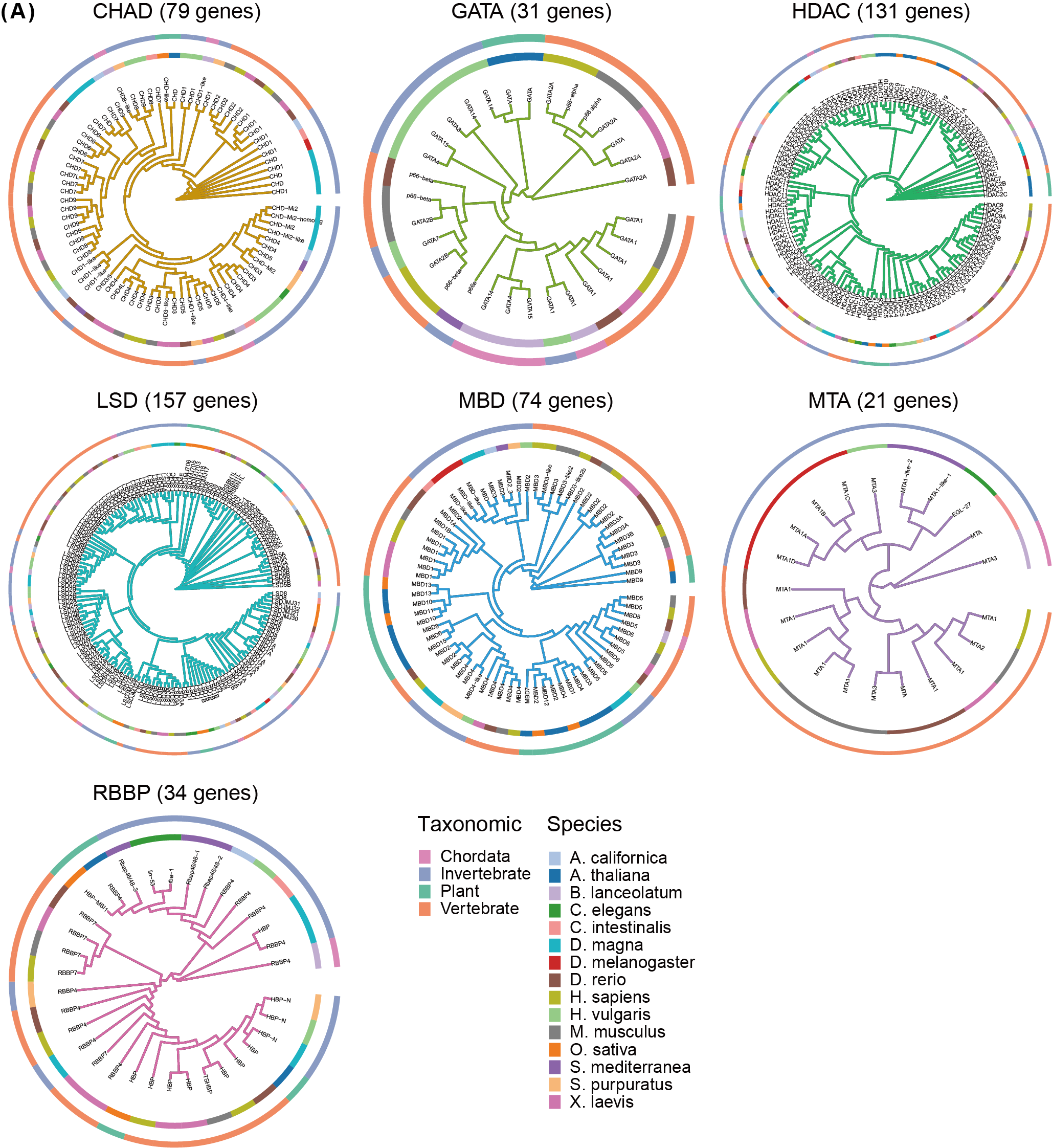


**Figure S1**. Maximum likelihood (ML) phylogenetic tree of NuRD complex candidate genes. **(A)** Maximum likelihood (ML) phylogenetic tree showing the evolutionary relationships of seven gene families (comprising 11 genes) associated with the NURD complex across 15 species.


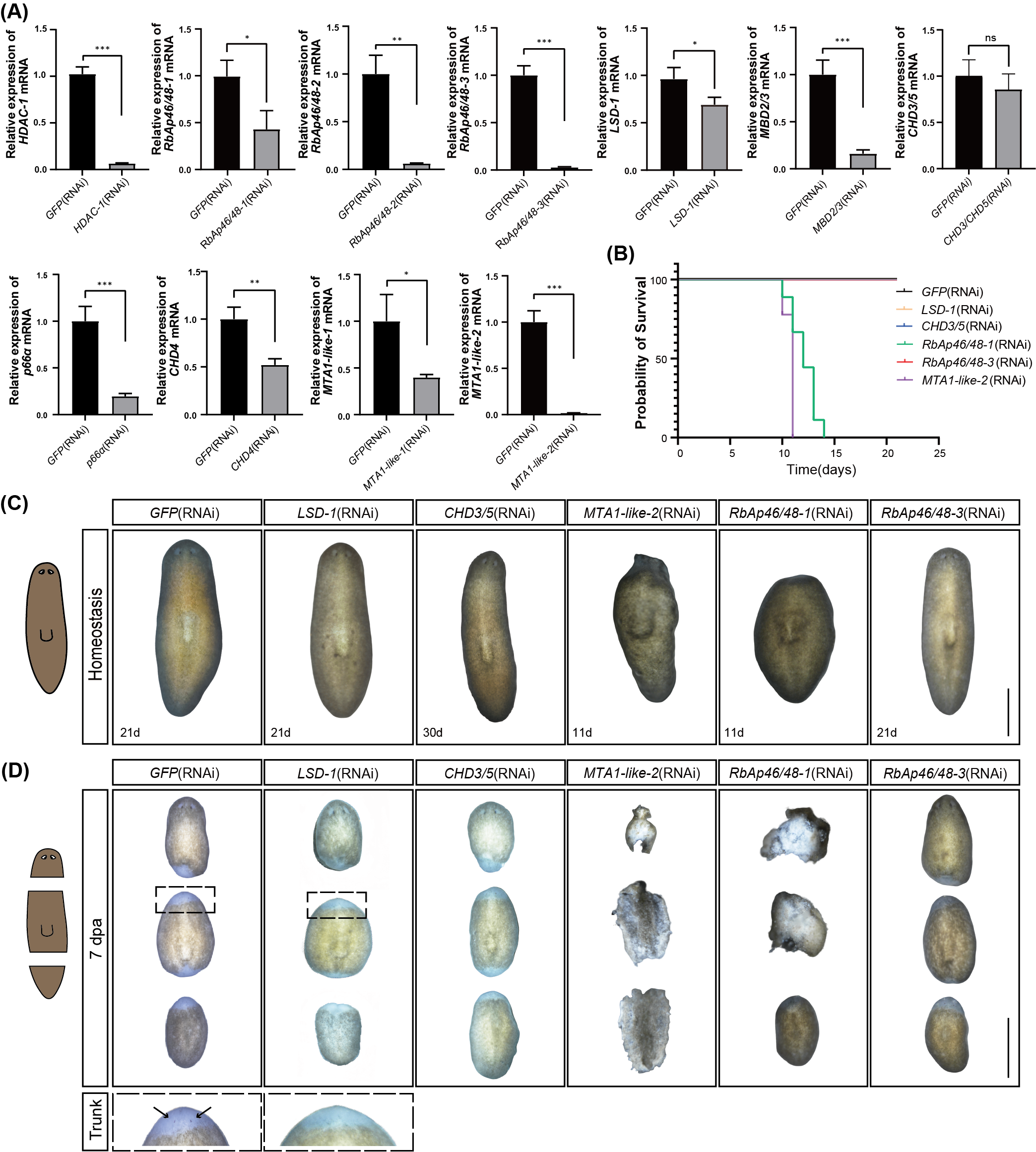


**Figure S2.** RNAi phenotypes of core NuRD complex genes on planarian homeostasis and regeneration. **(A)** qRT-PCR analyses of gene knockdown efficiency of NuRD complex genes by RNAi. Error bars represent mean values ± SD (*n* = 3). T-tests were used for statistical comparisons, ns denotes not significant, **p* < 0.05, ***p* < 0.01, ****p* < 0.001. **(B)** Survival curves of planarians after NuRD complex genes RNAi treatments (*n* = 18). **(C)** Effects of NuRD complex gene silence on the homeostasis of planarians (*n* = 18). Scale bars: 1000 µm. **(D)** Effects of NuRD complex gene silence on the regeneration of planarians (*n* = 18). Scale bars: 1000 µm. The dashed boxes contain the amplified images of eye regeneration.


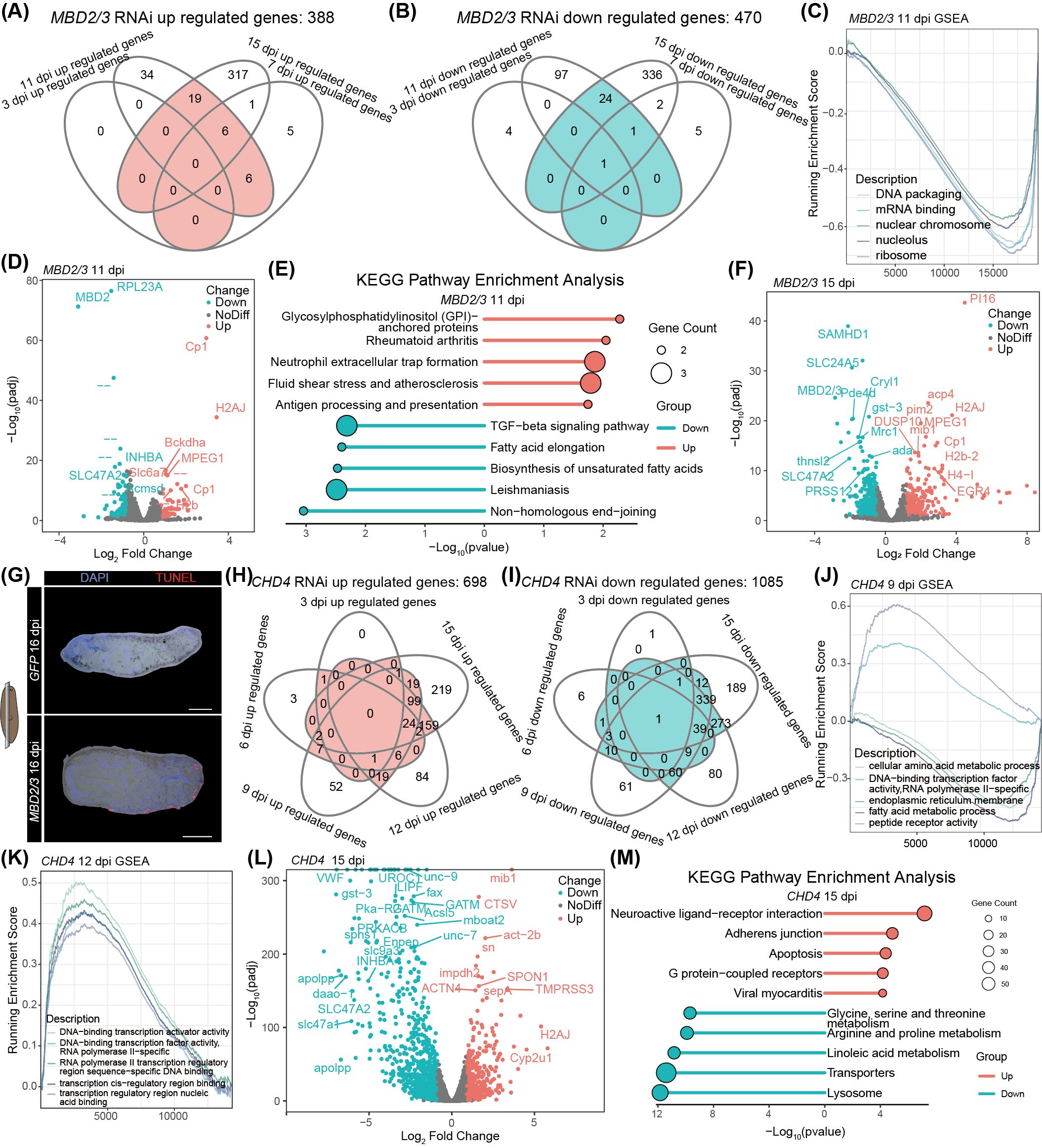


**Figure S3.** Transcriptomic data analyses of the effects of *MBD2/3* or *CHD4* RNAi in planarians. **(A-B)** Venn diagram showing the up **(A)** and down **(B)** regulated intersection genes at 3, 7, 11 and 15 dpi after *MBD2/3* RNAi treatment. **(C)** Gene set enrichment analyses at 11 dpi after *MBD2/3* depletion. **(D)** Volcano plot showing downregulated (blue) or upregulated (red) genes at 11 dpi after *MBD2/3* RNAi treatment. **(E)** KEGG pathway enrichment analyses of significantly differentially expressed genes at 11 dpi after *MBD2/3* RNAi treatment. **(F)** Volcano plot showing downregulated (blue) or upregulated (red) genes at 15 dpi after *MBD2/3* RNAi treatment. **(G)** TUNEL staining of paraffin-embedded tissue sections from *GFP* and *MBD2/3* RNAi treatment animals. Scale bar: 200 µm. **(H-I)** Venn diagram showing the up **(H)** and down **(I)** regulated intersection genes in animals at 3, 6, 9, 12 and 15 dpi after *CHD4* RNAi treatment. **(J-K)** Gene set enrichment analyses at 9 dpi **(J)** and 12 dpi **(K)** after *CHD4* depletion. **(L)** Volcano plot showing downregulated (blue) or upregulated (red) genes at 15 dpi after *CHD4* RNAi treatment. **(M)** KEGG pathway enrichment analyses of significantly differentially expressed genes at 15 dpi after *CHD4* RNAi treatment.


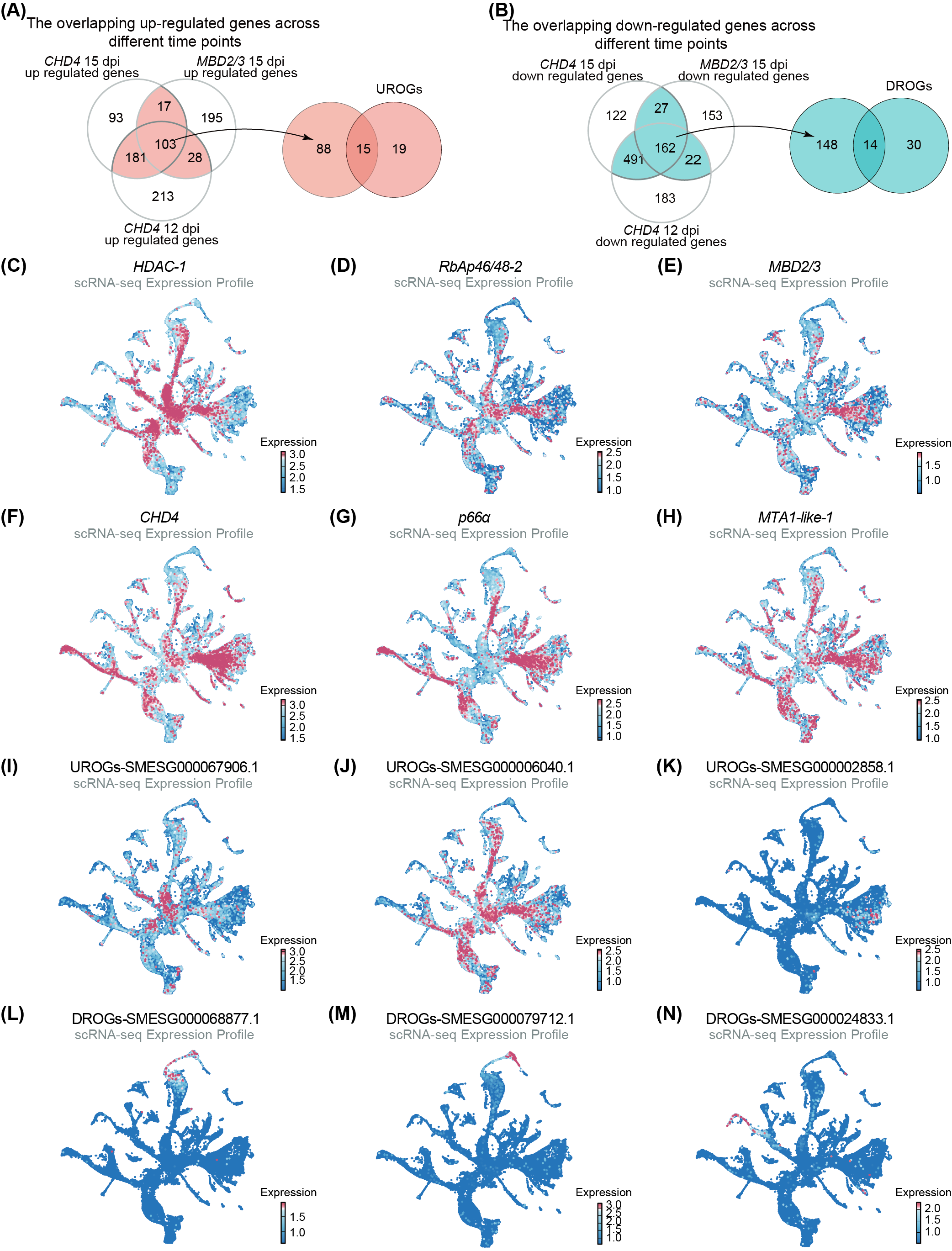


**Figure S4.** Core NuRD complex genes and their UROGs and DROGs in RNA-seq and scRNA-seq analyses of control and MBD2/3 and CHD4 knockdown planarians. **(A-B)** Venn diagram of upregulated **(A)** and downregulated **(B)** genes at 12 dpi and 15 dpi after *CHD4* RNAi treatment, and at 15 dpi after *MBD2/3* RNAi treatment. **(C-H)** Feature plots of individual core NuRD complex gene expression pattern. **(I-N)** Feature plot of three UROGs **(I-K)** and three DROGs **(L-N)** gene expression patterns.


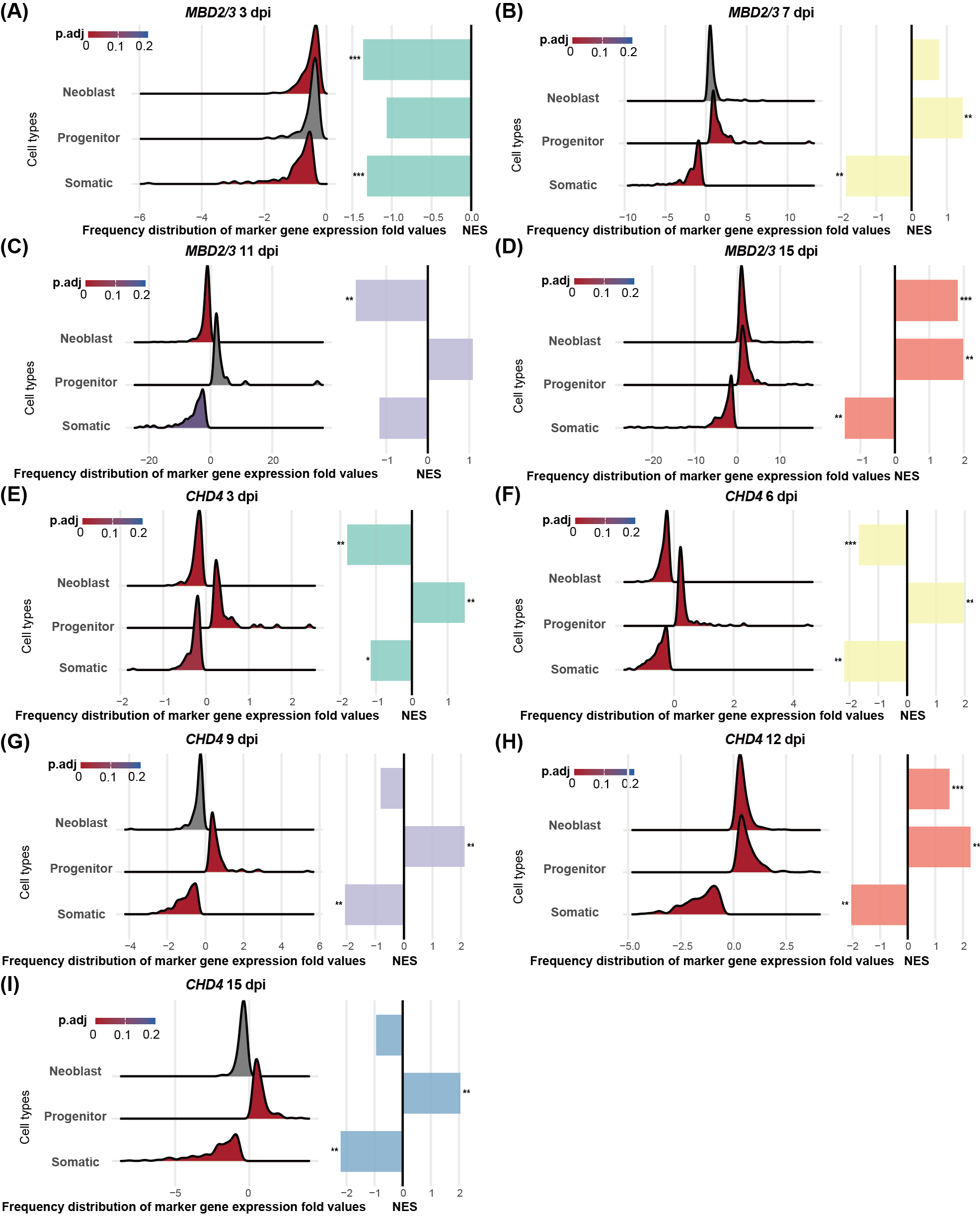


**Figure S5.** Frequency distribution of differentiation marker fold changes after *MBD2/3* or *CHD4* RNAi treatment. **(A-D)** Frequency distribution of differentiation marker fold change values at 3, 7, 11 and 15 dpi after *MBD2/3* depletion. **(E-I)** Frequency distribution of differentiation marker fold change values at 3, 6, 9, 12 and 15 dpi after *CHD4* depletion. The permutation test was used for statistical comparisons, **p* < 0.05, ***p* < 0.01, ****p* < 0.001.


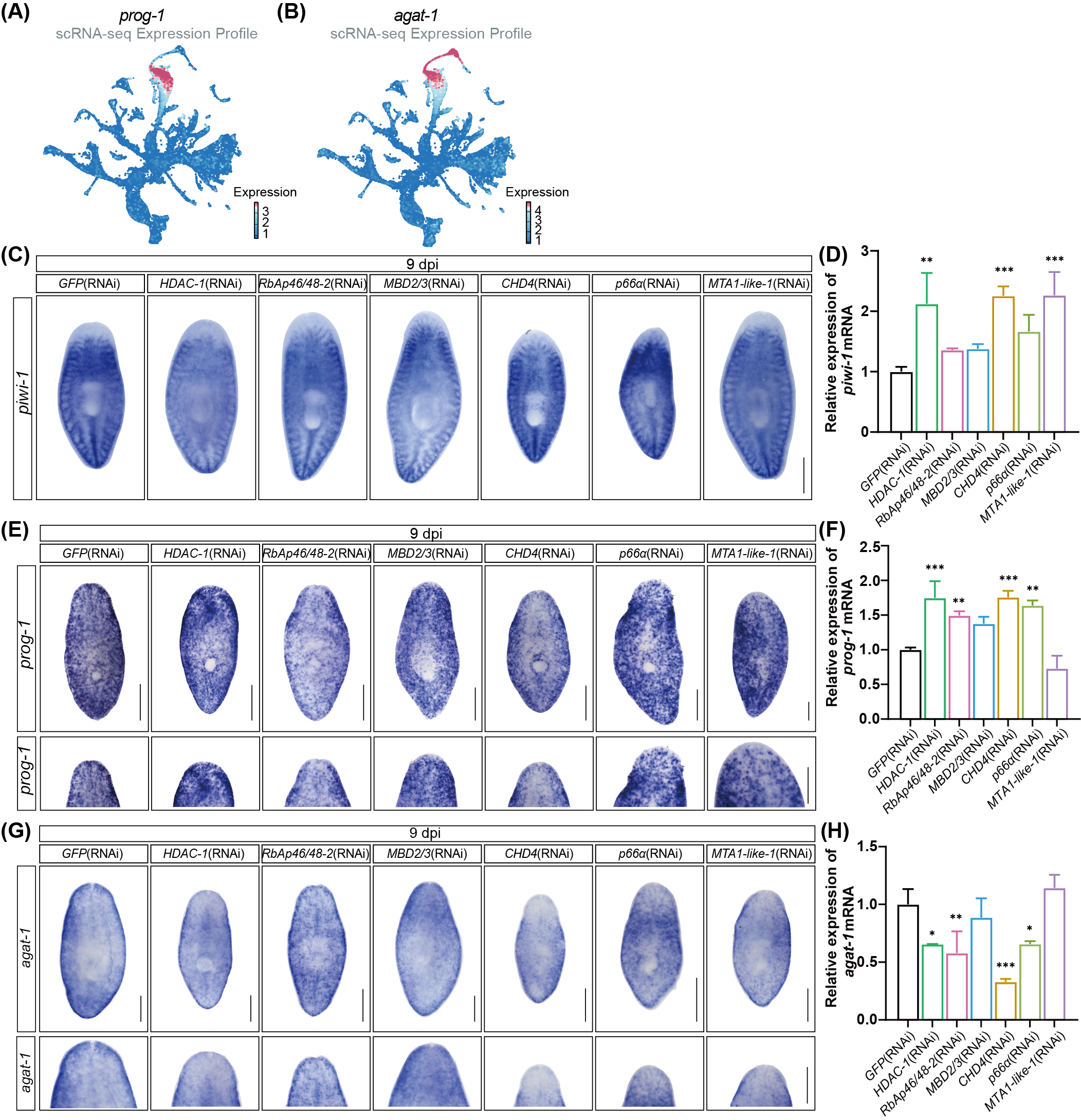


**Figure S6.** NuRD complex genes regulate the expression of cell differentiation marker genes. **(A-B)** Feature plots of the *prog-1* **(A)** and *agat-1* **(B)** expression. **(C)** WISH images of the *piwi-1* expression after the core NuRD complex gene RNAi treatment in planarians. **(D)** qRT-PCR quantification of *piwi-1* expression after RNAi treatment. Error bars represent mean values ± SD (*n* = 3). One-way ANOVA was used for statistical comparisons, ***p* < 0.01, ****p* < 0.001. **(E)** WISH images of the *prog-1* expression after NuRD complex core genes RNAi treatment. **(F)** qRT-PCR quantification of *prog-1* expression after NuRD complex genes RNAi treatment. Error bars represent mean values ± SD (*n* = 3). One-way ANOVA was used for statistical comparisons, ***p* < 0.01, ****p* < 0.001. **(G)** WISH images of the *agat-1* expression after NuRD complex gene RNAi treatment. **(H)** qRT-PCR quantification of *agat-1* expression after NuRD complex genes RNAi treatment. Scale bars: 500 µm. One-way ANOVA was used for statistical comparisons, **p* < 0.05, ***p* < 0.01, ****p* < 0.001.


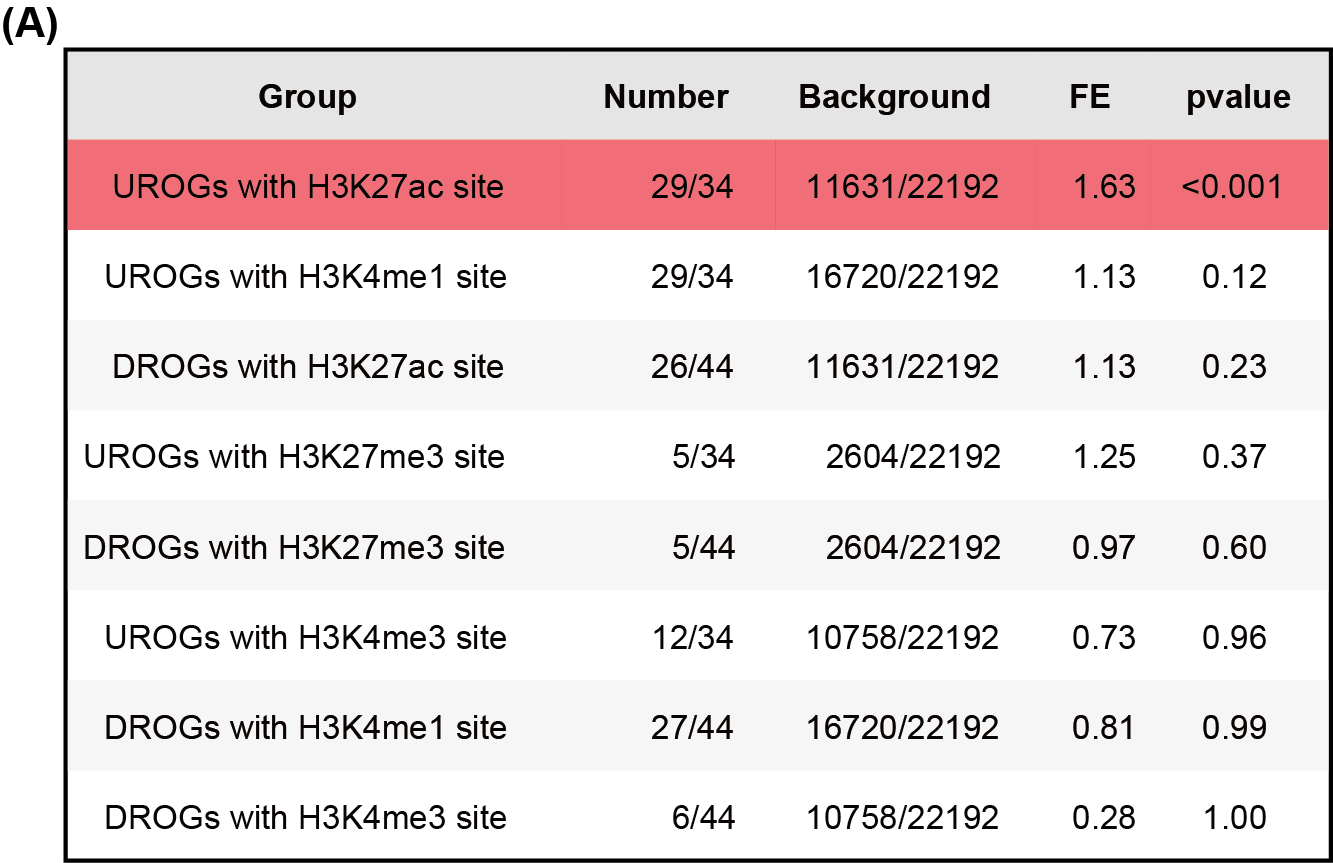


**Figure S7**. NuRD complex genes regulate cell differentiation through histone deacetylation. **(A)** Table showing hypergeometric distribution analyses of 34 UROGs and 44 DROGs with H3K27ac, H3K4me1, H3K4me3, and H3K27me3 sites. One-way ANOVA was used for statistical comparisons.
